# Mixed Method Research on Football Coaches’ Competitive Behavior

**DOI:** 10.3389/fpsyg.2021.705557

**Published:** 2021-07-13

**Authors:** José Rodrigues, Filipe Rodrigues, Rui Resende, Mário Espada, Fernando Santos

**Affiliations:** ^1^Sport Science School of Rio Maior, Polytechnic Institute of Santarém, Rio Maior, Portugal; ^2^Life Quality Research Centre, Rio Maior, Portugal; ^3^Sport and Physical Education School, University Institute of Maia, Maia, Portugal; ^4^Higher School of Education, Science, and Technology, Polytechnic Institute of Setúbal, Setúbal, Portugal

**Keywords:** mixed methods, coach behavior, competition, football, observation

## Abstract

**Objective:**

The purpose of this study was to present the reliability of three validated measures, namely the System of Analysis of Instruction in Competition, the Questionnaire on Coach Instructional Behavior Expectations, and the Questionnaire on Coach Instructional Behavior Perception that could be used in a mix-method approach.

**Methods:**

Three instruments underwent a robust process of construct and reliability analysis. Inter− and intra-observer reliability was tested for the observational instrument using *Cohen’s k*-agreement measure. Reliability values above 0.85 were considered as a good agreement between and within observers. To verify the internal consistency of the questionnaires, the correlation coefficients were considered.

**Results:**

The results related to intra-observer and inter-observer reliability showed that intra-observer reliability *k*-agreement values ranged between 0.912 and 1 for observer 1, and 0.82 and 1 for observer 2. For inter-observer reliability, *k*-agreement values ranged between 0.885 and 1 between observers. Thus, values for reliability are above acceptable. The correlation coefficient values recorded for the questionnaires on instruction expectations in the competitive moment were above 0.82 and significant (*p* < 0.05), and for the questionnaire on instruction perception in competition above 0.88 and significant (*p* < 0.05). The pilot study showed some divergent results across expectations, behavior during competition, and perception about the instruction behavior.

**Conclusion:**

The observational system and the expectations and perceptions questionnaires, used in a complementary way, can be considered as a mix-method approach for studies aiming to examine coaches’ competitive behavior.

## Introduction

Sport coaches play a key role in designing training sessions. In addition, they decisively contribute to the technical, tactical, physical, and psychological aspects of the players and the team overall during competition (Pesca et al., 2017). The coach–athlete relationship is a crucial aspect of the coaching process and the coaching type adopted by the coaches is capable of influencing expected results (Choi et al., 2020). Specifically, how coaches prepare working strategies and how the team is oriented from a technical point-of-view is determinant of increased athlete and team confidence and performance (Almeida et al., 2019; Keatlholetswe and Malete, 2019). In this regard, the coach needs to master communication skills since it has been reported previously to be associated with athlete and team accomplishments (Kim and Park, 2020; Moll and Davies, 2021). Coaches’ ability to communicate effectively is critical since all tasks related to athlete top performance requires high communication skills from coaches (Gomes et al., 2020).

Several studies have been developed aiming to understand coaching instructions (Smith and Cushion, 2006; Santos and Rodrigues, 2008; Santos et al., 2012, 2014b) and coaching feedback (Mason et al., 2020) in competitive moments. However, Hague et al. (2021) have indicated that there is still a need for more research related to the technical information inherent in sport coaching. Thus, how coaches communicate feedback and technical strategies as a mean to maximize athlete’s abilities is a key aspect that needs more investigation (Kim and Park, 2020). The coaching behavior is a combination of previously made decisions and reflexive thinking (Moreno and Alvarez, 2004). The perception of coaches about how they communicate can be important for a reflection on the strategies to be used in future competitive moments (Hague et al., 2021). The ability coaches perceive and analyze their instruction behavior following a reflective thinking is of enormous value for the development of learning experiences and increase quality of coaching behavior (Araya et al., 2015; Keatlholetswe and Malete, 2019).

Systematic observation of coaching behavior allows for researchers and practitioners to collect quality information related to the coach–athlete relationship in both training and competitive moments as a mean to increase performance (McKenzie and van der Mars, 2015). A systematic review conducted by Cope et al. (2017), aiming to provide an update of systematic observation methods in coaching research, identified 26 studies using different measures of coaching behavior. Cope et al. (2017) urged researchers to adopt a more vital approach when adopting a systematic observation method. The same authors offer a clearer rationale for the systematic observation instruments to be employed on the assessment of coaching behavior and suggest the use of a mixed methods approach. It is relevant for research purpose and coach education to evaluate the coach–athlete relationship aiming to define better coaching profiles and enable the integration of better communication behaviors in coaching courses. In addition, systematic observation enables the assessment on how coaches perform (Cope et al., 2017) allowing them to analyze their own behavior through a systematized data collection and analysis (Anguera et al., 2018a). Systematic observation is considered by itself a mixed method since it contains a qualitative (e.g., resulting from the codification of observed behaviors) and integrated quantitative (e.g., behaviors duration and frequency) data (Anguera et al., 2018a, b). Specifically, the use of T-*patterns* analysis (Santos et al., 2014a) or sequential analysis (García-Fariña et al., 2018) provides robustness to the integration of quantitative and qualitative data considering the mixed method type of analysis (Anguera et al., 2018b).

Considering the added value of using systematic observation (i.e., indirect observation) such as mixed method approaches, it is also possible to develop questionnaires (multi-methods) collecting data on the variables of interest, for example, the behaviors displayed by coaches during competition. Additionally, coach expectations and perceptions about their behavior are both crucial aspects that can be measured using mixed method type of investigation.

**FIGURE 1:**
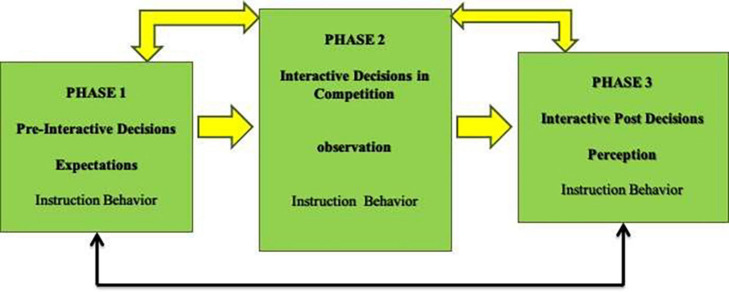
Coach behavior analysis model.

Bearing in mind the importance of coaching behavior and its implications on athlete and overall team performance, this study provides results from the examination of three instruments regarding the assessment of expectations, behavior, and perception data related to the instruction behavior during competition. These instruments provide information that is aggregated according to the three steps model of coaches’ decision-making related to tactics (Cloes et al., 2001). This model defines steps related to tactical decision-making of coaches in competition: (a) pre-interactive decisions (before the competition), (b) interactive decisions (during the competition), and (c) post-interactive decisions (after the competition). In exploring the factors inherent in each of the instruments under analysis, practitioners and coaches can assess coach preparations (expectations) and reflections (perception) related to instructional behavior, as well as the relationship between the three moments (before, during, and after the competition). As result, researchers are more prone to understand coaches’ communication strategies and how coaches prepare their interventions during competitive situations and how they can enhance coaching behaviors (Araya et al., 2015; Keatlholetswe and Malete, 2019; Kim and Park, 2020; Hague et al., 2021). In addition, training programs are re-evaluated according to coaching expectations and reflections for coaches to develop more effective instructional behaviors in the competition team direction (Moreno et al., 2007). In Figure 1, a behavior analysis model of the football coaching behavior during competitive moments is presented according to several authors (Santos et al., 2016, 2019).

Previous studies have found inconsistencies across expectations, perception, and instruction behavior in competition (Santos et al., 2016, 2019). This demonstrates that more research is needed to provide coaches the necessary tools to help coaching interventions as well as to optimize instruction behaviors. It is possible to verify that the coach’s instructional behavior is still strongly rooted in a traditional view of actions and behavior prescription (Oliva et al., 2010; Santos et al., 2012; 2014b), limiting the decision-making of the young players at competitive moments. According to existing limitations and gaps in the literature, the purpose of this study was to present the reliability of three validated measures, namely the System of Analysis of Instruction in Competition, the Questionnaire on Coach Instructional Behavior Expectations, and the Questionnaire on Coach Instructional Behavior Perception that could be used in a mix-method approach.

## Materials and Methods

### Participants

Participants were 16 coaches (male = 16) aged between 35 and 50 years (*M* = 42.5, SD = 5.59), who participated in the different stages of the construction and validation of the instruments. Regarding their level of education, 16 (100%) had a bachelors’ degree in sport and exercise science, and 16 (100%) had completed certified postgraduate courses for professional coaching. Coaches were licensed professionals, having a mean work experience of 14.5 years (SD = 6.18). Participants were recruited between September 2011 and January 2012. The participants were professional coaches competing in Portuguese national championships. These coaches worked in clubs of the Santarém Football Association (*n* = 6), Lisbon Football Association (*n* = 6), and Leiria Football Association (*n* = 4).

Potential participants who agreed to participate voluntarily in this study had to comply with the following inclusion criteria: equal or more than 5 years of coaching experience with young people (Smith and Cushion, 2006); competing in national championships (Côté and Salmela, 1996; Smith and Cushion, 2006); have a degree in physical education and/or sports; and have a professional title of sports coach recognized by the certified institutions (Potrac et al., 2002). The coaches were selected according to the characteristics mentioned above and were invited to participate in this research voluntarily.

Before data collection, the study was reviewed and approved by the Ethical Committee. Data collection procedures were carried out according to the Helsinki declaration and its later amendments, and all participants completed an informed consent form. Subsequently, the study’s objectives and data collection procedures were explained to several football clubs. After approval, coaches were contacted day and were asked to participate voluntarily in this study. We specifically asked for permission to collect data regarding their behaviors during coaching, reinforcing anonymity, and confidentiality. For the second phase, coaches who accepted to partake to this study signed an informed consent form, and meetings were scheduled to clarify the objectives and methodological procedures to be developed for data collection.

It has previously been recommended that qualitative studies require a minimum sample size of at least 12 to reach data saturation. Therefore, a sample of 14 was deemed sufficient for the qualitative analysis. In this study we did not conducted traditional factor analyses for factor validity of the quantitative measures. We focused solely on the reliability of the measures, since they had been validated previously (Santos et al., 2016, 2019).

### Instruments

#### Phases for the Development of the Observational Instrument

Data on the behavior of instruction during competition were collected using observational instruments (Santos and Rodrigues, 2008). The objective was to code their instructional behaviors when interacting with the team during the competitive moment. For this purpose, the Instruction Analysis System in Competition (Santos and Rodrigues, 2008) was used to measure coaching behavior. This observational instrument has been tested and used in previous studies (e.g., Santos et al., 2012, 2014b), when examining behavioral instructions provided by senior and youth coaches. This instrument consist of four criteria, namely: (a) objective, (b) form, (c) direction, and (d) content. These criterions are grouped by 21 categories and 28 subcategories (for details see Table 1). For construct reliability and validity, the observational instrument was put to test in three stages.

**TABLE 1:** Instruction analysis system during competition.

Objective	Form	Direction
**Evaluative + (EV+)**	**Hearing (H)**	**Athlete (ATL)**
**Evaluative** – **(EV−)**	**Visual (VIS)**	**Substitute Athlete (SA)**
**Descriptive (DES)**	**Hearing-visual (H-VIS)**	**Group (GRO):**
**Prescriptive (PRE)**		Defenses Group (DG)
**Interrogative (INT)**		Midfielders Group (MG)
**Affectivity + (AF+)**		Forward Group (FG)
**Affectivity** – **(AF−)**		Substitutes Group (SG)
		Team (T)

**Content**		

**Technique (TEC):**	**Psychological (PSY):**	**Physical (FIS):**
Offensive Techniques (OFTE)	Game Rhythm (PGR)	Resistance (PRES)
Defensive Techniques (DEFTE)	Confidence (PC)	Execution speed (PES)
**Tactic (TAT):**	Pressure effectiveness (PPE)	Displacement speed (PDS)
Game System (TAGS)	Attention (PAT)	Reaction speed (PRS)
Game Methods (TAGM)	Concentration (PCO)	Strength (PS)
Tactical Schemes (TATS)	Combative pressure (PPC)	Warm up (PWU)
Game Principles (TAGP)	Adversity resistance (PAR)	**Opponent’s Team (Opp T)**
Functions/Missions (TAFUNC)	Responsibility (PRE)	**Referees Team (RT)**
Combinations (TACOMB)		**No content (N/C)**
General Effectiveness (TAGE)		**Indeterminate (IND)**

*Caps and bold = criteria; bold = categories; not bold = subcategories.*

##### Stage 1 – literature review for the observational instrument construction

In the first phase, the observational instrument was developed based on existing literature (Pulido et al., 2019). A literature review was performed exploring available measures of coaching behavior. The observational instrument was created based on the Observation System of Instruction in Volleyball (Pina and Rodrigues, 1994). Adaptations were made for the football context under analysis regarding categories and subcategories. Through the review of the specific literature of football, the categories and subcategories related to the content of the instruction were listed.

##### Stage 2 – expert panel evaluation

After the development of the observational instrument and after guaranteeing the completeness and exclusivity of the category system, the measure was analyzed by a panel of experts to ensure content validity following Pulido et al. (2019) recommendations.

##### Stage 3 – reliability analysis

The final version of the observational instrument was presented for reliability analysis. In this stage, intra− and inter-observer reliability was tested considering evidenced-based recommendations (Brewer and Jones, 2002). The reliability of observers is one of the utmost crucial conditions in behavioral observation studies for data quality (Blanco-Villaseñor et al., 2014). The instrument reliability is assumed when it presents few errors, demonstrates stability, consistency, and dependence on the result of the observations made. For this study, the stability of intra-sessional measurement was analyzed through a two-facet design (categories and observers = C/O) as proposed by Blanco-Villaseñor et al. (2014).

#### Stages for the Development of the Questionnaires

The Coach Instructional Behavior Expectations Questionnaire and the Coach Instructional Behavior Perception Questionnaire (Santos et al., 2016, 2019) were used to measure coaches’ expectations and perceptions about their instructional behaviors. Each questionnaire is composed of 20 questions and has a relationship with the categories and subcategories of the observational instrument previously mentioned. Answers are provided using a Likert scale anchored between 1 (nothing) and 5 (very).

The validation of the questionnaires went through a process composed of five stages as proposed by Brislin (1980), namely: (1) preliminary study for the construction of the first version of the questionnaires; (2) creation of the first version of the questionnaires; (3) expert assessment and validation of the questionnaires; (4) reliability analysis of the questionnaires; and (5) final version and pilot study.

##### Stage 1 – construction of the questionnaire through literature review

In the first stage, all variables to be considered were proposed and listed aiming that the questionnaire was following the objectives and hypotheses of the study. This procedure was conducted considering the Instruction Analysis System in Competition. This first phase also considered the questionnaire on coach instruction expectations during lecture and competition (Santos and Rodrigues, 2008), a validated instrument in senior coaches. The construction of the first version of the questionnaire was performed according to the type of the desired answer and considering data treatment. As such, we choose answers to the questions through a Likert scale, a scale widely used in sports coaching research (Rhind et al., 2014), which allowed verifying expectations and perceptions about the amount of instruction.

##### Stage 2 – expert feedback

In the second stage, the questionnaires were evaluated by a panel of experienced coaches in youth training and graduated in physical education and sports. As previously mentioned, the construction of the questionnaires was based on existing questionnaires that had been validated for application in senior coaches (Santos and Rodrigues, 2008) and now adapted for coaches who train young athletes. According to Rhind et al. (2014), the application of existing questionnaires in other populations from the original validation should be carried out by structurally examining the validity of the instrument in the context where it will be applied.

##### Stage 3 – validation by experts

In the third stage, the questionnaires were submitted to content validation by a panel of ten experts according to proposed procedures (Cid et al., 2012). The panel consisted of six Ph.D. researchers and four youth football coaches, of which the last two were graduates in physical education and sports and two certified masters in sport training. The experts were asked for a qualitative evaluation of the questionnaires and all the comments for improvement were recorded (Quinaud et al., 2018). According to the request, the experts reported that the questions were overall following the variables that are intended to measure the level of expectations and perception (categories of the observational instrument) and suggested some changes for clarifying content, objective, and enabling the introduction of practical examples in the questions. After introducing the proposed changes provided by the experts, the questionnaires were applied in a pilot study. This stage was performed to test all procedures in the application of the used instruments (observational instrument and questionnaires). In the end, the participants in the pilot study reported that they had no problem or doubts answering the questionnaire.

##### Stage 4 – reliability analysis

In the fourth stage, reliability analysis was performed. Reliability analysis ensures that the instrument is consistent (Cid et al., 2012). For this study, consistency was verified by the equivalence of the answers given to two versions of the same question (Hill and Hill, 2009). Therefore, correlations were calculated for the reliability coefficient estimation. The questionnaires were applied to five coaches, who did not participate in the previous stages. The five coaches answered the questionnaires within the context in which this investigation was carried out. At the beginning of a competitive game, the coaches answered the questionnaire about the instruction expectations in competition and at the final of the game coaches answered to the perception questionnaire about the instruction behavior during competition.

##### Stage 5 – construction of the final version of the questionnaires

In the fifth and final stage, the two questionnaires reached the final Portuguese version for youth athletes. Both measures of expectations and perception of behavioral instruction in the competition are displayed in Table 2.

**TABLE 2:** Coach expectations and coach perceptions of behavioral instructions questionnaires.

Coach Instructional Behavior Expectations Questionnaire	Coach Instructional Behavior Perception Questionnaire
1 – Há treinadores que emitem a informação com o **objetivo avaliativo positivo** (ex.: “Bem jogado”) Emite este tipo de Informação?	1 – Há treinadores que emitem a informação com o **objetivo avaliativo positivo** (ex.: “Bem jogado”) Emitiu este tipo de Informação?
2 – Há treinadores que emitem a informação com o **objetivo avaliativo negativo** (ex.: “Não é isso”) Emite este tipo de Informação?	2 – Há treinadores que emitem a informação com o **objetivo avaliativo negativo** (ex.: “Não é isso”) Emitiu este tipo de Informação?
3 – Há treinadores que emitem a informação com **objetivo descritivo**, ou seja, descrevem aquilo que o jogador fez ou está a fazer (ex.: “eles fazem uma boa circulação de bola”) Emite este tipo de informação?	3 – Há treinadores que emitem a informação com **objetivo descritivo**, ou seja, descrevem aquilo que o jogador fez ou está a fazer (ex.: “eles fazem uma boa circulação de bola”) Emitiu este tipo de informação?
4 – Há treinadores que emitem informação com o **objetivo prescritivo**, ou seja, prescrevem aquilo que o jogador deverá fazer futuramente (ex.: “deves executar o passe longo da seguinte forma…”). Emite este tipo de informação?	4 – Há treinadores que emitem informação com o **objetivo prescritivo**, ou seja, prescrevem aquilo que o jogador deverá fazer futuramente (ex.: “deves executar o passe longo da seguinte forma…”). Emitiu este tipo de informação?
5 – Os treinadores **colocam questões** ao jogador sobre o seu comportamento (ex.: “achas que estás a marcar bem o teu adversário?”). Emite este tipo de informação?	5 – Os treinadores **colocam questões** ao jogador sobre o seu comportamento (ex.: “achas que estás a marcar bem o teu adversário?”). Emitiu este tipo de informação?
6 – O treinador reage à prestação ou futura prestação do jogador **incentivando-o** (“muito bom”). Emite este tipo de informação?	6 – O treinador reage à prestação ou futura prestação do jogador **incentivando-o** (“muito bom”). Emitiu este tipo de informação?
7 – O treinador reage à prestação ou futura prestação do jogador **criticando-o simplesmente** (ex.: “não estás a jogar nada, qualquer outro fazia melhor que tu”). Emite este tipo de informação?	7 – O treinador reage à prestação ou futura prestação do jogador **criticando-o simplesmente** (ex.: “não estás a jogar nada, qualquer outro fazia melhor que tu”). Emitiu este tipo de informação?
8 – A informação que transmite tem uma forma exclusivamente **auditiva**, ou seja, comunica verbalmente?	8 – A informação que transmitiu tem uma forma exclusivamente **auditiva**, ou seja, comunicou verbalmente?
9 – A informação que transmite tem uma forma exclusivamente **visual**, ou seja, gestual?	9 – A informação que transmitiu tem uma forma exclusivamente **visual**, ou seja, gestual?
10 – A informação que transmite tem uma **forma auditiva-visual** (verbal e gestual)?	10 – A informação que transmitiu tem uma **forma auditiva-visual** (verbal e gestual)?
11 – A informação que transmitiu foi dirigida individualmente (para um jogador titular)?	11 – A informação que transmite é dirigida individualmente (para um jogador titular)?
12 – A informação que transmite é dirigida coletivamente (**para toda a equipa**)?	12 – A informação que transmitiu foi dirigida coletivamente (**para toda a equipa**)?
13 – A informação que transmite é dirigida para um grupo de jogadores (**conjunto de jogadores**)?	13 – A informação que transmitiu foi dirigida para um grupo de jogadores (**conjunto de jogadores**)?
13 (a) A informação que transmite é dirigida para o **grupo de defesas**?	13 (a) A informação que transmitiu foi dirigida para o **grupo de defesas**?
13 (b) A informação que transmite é dirigida para o **grupo de médios**?	13 (b) A informação que transmitiu foi dirigida para o **grupo de médios**?
13 (c) A informação que transmite é dirigida para o **grupo de avançados**?	13 (c) A informação que transmitiu foi dirigida para o **grupo de avançados**?
13 (d) A informação que transmite é dirigida para o **grupo de suplentes**?	13 (d) A informação que transmitiu foi dirigida para o **grupo de suplentes**?
14 – A informação que transmite é dirigida para um **suplente**?	14 – A informação que transmitiu foi dirigida para um **suplente**?
15 – A informação que transmite apresenta um **conteúdo técnico**, ou seja, sustentada na execução motora dos diferentes comportamentos dos jogadores? (ex.: “recebe a bola com a parte interna do pé”)	15 – A informação que transmitiu apresentou um **conteúdo técnico**, ou seja, sustentada na execução motora dos diferentes comportamentos dos jogadores? (ex.: “recebe a bola com a parte interna do pé”)
15 (a) A informação de **conteúdo técnico** que transmite é relativa aos **comportamentos ofensivos**, ou seja, quando a equipa tem a posse da bola (receção, passe, remate, condução, etc.)?	15 (a) A informação de **conteúdo técnico** que transmitiu foi relativa aos **comportamentos ofensivos**, ou seja, quando a equipa tem a posse da bola (receção, passe, remate, condução, etc.)?
15 (b) A informação de **conteúdo técnico** que transmite é relativa aos **comportamentos defensivos**, ou seja, quando a equipa não tem a posse da bola (interceção, desarme, posição de base defensiva)?	15 (b) A informação de **conteúdo técnico** que transmitiu foi relativa aos **comportamentos defensivos**, ou seja, quando a equipa não tem a posse da bola (interceção, desarme, posição de base defensiva)?
16 – A informação que transmite apresenta um **conteúdo tático**, ou seja, baseada na capacidade individual e coletiva que os jogadores têm para resolver eficazmente as diferentes situações de jogo? (ex.: “a última linha defensiva tem de jogar o mais à frente possível”)	16 – A informação que transmitiu apresentou um **conteúdo tático**, ou seja, baseada na capacidade individual e coletiva que os jogadores têm para resolver eficazmente as diferentes situações de jogo? (ex.: “a última linha defensiva tem de jogar o mais à frente possível”)
16 (a) A informação de **conteúdo tático** que transmite é relativa ao **sistema de jogo**, ou seja, à forma como a equipa se distribui dentro do campo? (ex.: “Jogamos com 2 avançados”)	16 (a) A informação de **conteúdo tático** que transmitiu foi relativa ao **sistema de jogo**, ou seja, à forma como a equipa se distribui dentro do campo? (ex.: “Jogamos com 2 avançados”)
16 (b) A informação de **conteúdo tático** que transmite é relativa aos **métodos de jogo**, ou seja, à forma como a equipa organiza o processo ofensivo e defensivo? (ex.: “Mantém a posse de bola” ou “defende à zona”)	16 (b) A informação de **conteúdo tático** que transmitiu foi relativa aos **métodos de jogo**, ou seja, à forma como a equipa organiza o processo ofensivo e defensivo? (ex.: “Mantém a posse de bola” ou “defende à zona”)
16 (c) A informação de **conteúdo tático** que transmite é relativa aos **esquemas táticos**, ou seja, à forma que a equipa organiza as situações de bola parada, no plano ofensivo e defensivo? (livres diretos e indiretos, pontapés de canto e lançamentos de linha lateral)	16 (c) A informação de **conteúdo tático** que transmitiu foi relativa aos **esquemas táticos**, ou seja, à forma que a equipa organiza as situações de bola parada, no plano ofensivo e defensivo? (livres diretos e indiretos, pontapés de canto e lançamentos de linha lateral)
16 (d) A informação de **conteúdo tático** que transmite é relativa aos **princípios específicos do jogo** (ofensivos – progressão, cobertura, mobilidade e espaço; defensivos – contenção, cobertura, equilíbrio e concentração)?	16 (d) A informação de **conteúdo tático** que transmitiu foi relativa aos **princípios específicos do jogo** (ofensivos – progressão, cobertura, mobilidade e espaço; defensivos – contenção, cobertura, equilíbrio e concentração)?
16 (e) A informação de **conteúdo tático** que transmite é relativa às **missões/funções** ofensivas e defensivas dos diferentes jogadores que compõem a equipa? (ex.: “jogas a defesa central marcando individualmente o avançado”)	16 (e) A informação de **conteúdo tático** que transmitiu foi relativa às **missões/funções** ofensivas e defensivas dos diferentes jogadores que compõem a equipa? (ex.: “jogas a defesa central marcando individualmente o avançado”)
16 (f) A informação de **conteúdo tático** que transmite é relativa às **combinações e/ou circulações táticas ofensivas**? (ex.: “executem mais tabelinhas”)	16 (f) A informação de **conteúdo tático** que transmitiu foi relativa às **combinações e/ou circulações táticas ofensivas**? (ex.: “executem mais tabelinhas”)
16 (g) A informação de **conteúdo tático** que transmite é relativa à **eficácia da equipa a nível geral?** (ex.: “joga no chão”)	16 (g) A informação de **conteúdo tático** que transmitiu foi relativa à **eficácia da equipa a nível geral?** (ex.: “joga no chão”)
17 – A informação que transmite apresenta um **conteúdo psicológico**, ou seja, baseada nas competências psicológicas fundamentais para a obtenção de melhor rendimento? (ex.: “temos de estar concentrados” ou “vamos ter confiança”)	17 – A informação que transmitiu apresentou um **conteúdo psicológico**, ou seja, baseada nas competências psicológicas fundamentais para a obtenção de melhor rendimento? (ex.: “temos de estar concentrados” ou “vamos ter confiança”)
17 (a) A informação de **conteúdo psicológico** que transmite procura aumentar o **ritmo/intensidade de jogo**? (ex.: “aumenta o ritmo”)	17 (a) A informação de **conteúdo psicológico** que transmitiu procurou aumentar o **ritmo/intensidade de jogo**? (ex.: “aumenta o ritmo”)
17 (b) A informação de **conteúdo psicológico** que transmite visa promover a **confiança** nos jogadores? (ex.: “tu a seguir fazes golo”)	17 (b) A informação de **conteúdo psicológico** que transmitiu visou promover a **confiança** nos jogadores? (ex.: “tu a seguir fazes golo”)
17 (c) A informação de **conteúdo psicológico** que transmite visa uma maior **eficácia do jogo**? (ex.: “Vamos, equipa”)	17 (c) A informação de **conteúdo psicológico** que transmitiu visou uma maior **eficácia do jogo**? (ex.: “Vamos, equipa”)
17 (d) A informação de **conteúdo psicológico** que transmite visa solicitar aos jogadores mais **atenção** a um determinado aspeto do jogo? (ex.: “Atenção à marcação a esse jogador”)	17 (d) A informação de **conteúdo psicológico** que transmitiu visou solicitar aos jogadores mais **atenção** a um determinado aspeto do jogo? (ex.: “Atenção à marcação a esse jogador”)
17 (e) A informação de **conteúdo psicológico** que transmite visa solicitar aos jogadores máxima **concentração** em determinadas situações de jogo? (ex.: “concentração nas bolas paradas”)	17 (e) A informação de **conteúdo psicológico** que transmitiu visou solicitar aos jogadores máxima **concentração** em determinadas situações de jogo? (ex.: “concentração nas bolas paradas”)
17 (f) A informação de **conteúdo psicológico** que transmite visa incentivar os jogadores, no sentido dum maior nível de **combatividade** no jogo? (ex.: “Equipa, vamos ser decididos na disputa da bola”)	17 (f) A informação de **conteúdo psicológico** que transmitiu visou incentivar os jogadores, no sentido dum maior nível de **combatividade** no jogo? (ex.: “Equipa, vamos ser decididos na disputa da bola”)
17 (h) A informação de **conteúdo psicológico** que transmite visa apelar à **responsabilidade** individual ou coletiva em jogo? (ex.: “Vamos ter responsabilidade”)	17 (h) A informação de **conteúdo psicológico** que transmitiu visou apelar à **responsabilidade** individual ou coletiva em jogo? (ex.: “Vamos ter responsabilidade”)
17 (g) A informação de **conteúdo psicológico** que transmite visa apelar a uma **resistência às adversidades** do jogo? (ex.: “Equipa não baixa a cabeça”)	17 (g) A informação de **conteúdo psicológico** que transmitiu visou apelar a uma **resistência às adversidades** do jogo? (ex.: “Equipa não baixa a cabeça”)
18 – A informação que transmite apresenta um **conteúdo físico**, ou seja, sustentada nas exigências físicas se um determinado comportamento individual ou coletivo? (ex.: “lança (ou remata) com mais força”)	18 – A informação que transmitiu apresentou um **conteúdo físico**, ou seja, sustentada nas exigências físicas se um determinado comportamento individual ou coletivo? (ex.: “lança (ou remata) com mais força”)
18 (a) A informação de **conteúdo físico** que transmite é relativa à **resistência**? (ex.: …, resiste ao esforço)	18 (a) A informação de **conteúdo físico** que transmitiu foi relativa à **resistência**? (ex.: …, resiste ao esforço)
18 (b) A informação de **conteúdo físico** que transmite é relativa à **velocidade de execução**? (ex.: …, executa mais rápido)	18 (b) A informação de **conteúdo físico** que transmitiu foi relativa à **velocidade de execução**? (ex.: …, executa mais rápido)
18 (c) A informação de **conteúdo físico** que transmite é relativa à **velocidade de deslocamento**? (ex.: …, mais rápido)	18 (c) A informação de **conteúdo físico** que transmitiu foi relativa à **velocidade de deslocamento**? (ex.: …, mais rápido)
18 (d) A informação de **conteúdo físico** que transmite é relativa à **velocidade de reação**? (ex.: reage rapidamente)	18 (d) A informação de **conteúdo físico** que transmitiu foi relativa à **velocidade de reação**? (ex.: reage rapidamente)
18 (e) A informação de **conteúdo físico** que transmite é relativa à **força**? (ex.: …, remata com força)	18 (e) A informação de **conteúdo físico** que transmitiu foi relativa à **força**? (ex.: …, remata com força)
18 (f) A informação de **conteúdo físico** que transmite é relativa ao **aquecimento**? (ex.: …, vai aquecer)	18 (f) A informação de **conteúdo físico** que transmitiu foi relativa ao **aquecimento**? (ex.: …, vai aquecer)
19 – A informação que transmite apresenta um conteúdo relativo à **equipa adversária**? (ex.: “O n^°^6 junta-se ao avançado”)	19 – A informação que transmitiu apresentou um conteúdo relativo à **equipa adversária**? (ex.: “O n^°^6 junta-se ao avançado”)
20 – A informação que transmite apresenta um conteúdo relativo à **equipa arbitragem**?	20 – A informação que transmitiu apresentou um conteúdo relativo à **equipa arbitragem**?

### Data Analysis

Cohen’s Kappa agreement measure for intra-observer and inter-observer reliability (Cohen, 1960) in this study using LINCE software (Gabin et al., 2012) was used. Scores ≥ 0.85 were indicative of acceptable inter-rater reliability (Altman, 1991). For the calculation of the value of *K* by category and in total, the existing functionality was used.

The reliability of the questionnaires was examined through temporal consistency analysis of the answers provided by the coaches. For this analysis bivariate correlation coefficients were considered, accepting significant *p*-value < 0.05. For these statistical procedures, the SPSS Statistics for Windows version 23.0 (SPSS Inc., Chicago, IL, United States) was considered.

## Results

The results related to intra-observer and inter-observer reliability for the observational instrument are displayed in Tables 3, 4. Table 3 shows that intra-observer reliability Kappa values ranged between 0.912 and 1 for observer 1, and 0.82 and 1 for observer 2. Table 4 shows that inter-observer reliability Kappa values ranged between 0.885 and 1 between observers. Thus, values for reliability are above acceptable.

**TABLE 3:** Intra-observer reliability of the observational system.

Criteria	Categories	Observer 1			Observer 2		
			
		1st Observation	2nd Observation	Kappa value	1st Observation	2nd Observation	Kappa value
Objective (+)	EV+	21	21	1.000	20	21	0.972
	EV−	2	2	1.000	2	2	1.000
	DES	6	6	1.000	5	7	0.826
	PRE	111	111	1.000	112	110	0.984
	INT	2	2	1.000	2	2	1.000
	AF+	4	4	1.000	5	4	0.885
	AF−	0	0	+++	0	0	+++
Form (+)	H	104	106	0.934	103	103	0.967
	VIS	0	0	+++	0	0	+++
	H-AUVIS	42	40	0.934	43	43	0.967
Direction (+)	ATL	118	118	0.978	118	119	0.978
	SA	1	1	1.000	1	1	1.000
	DG	2	2	1.000	2	2	1.000
	MG	2	2	1.000	2	2	1.000
	FG	0	0	+++	0	0	+++
	SG	4	4	1.000	4	4	1.000
	T	19	19	0.970	19	18	0.969
Content (+)	OFTE	6	6	1.000	6	6	1.000
	DEFTE	0	0	+++	0	0	+++
	TAGS	0	0	+++	0	0	+++
	TAGM	25	25	0.975	25	26	0.929
	TATS	32	33	0.941	33	32	0.941
	TAGP	3	3	1.000	3	3	1.000
	TAFUN	3	3	1.000	3	3	1.000
	TACOM	0	0	+++	0	0	+++
	TAGE	3	3	1.000	3	3	1.000
	PGR	2	2	1.000	2	2	1.000
	PC	0	0	+++	0	0	+++
	PPE	27	26	0.912	26	26	0.906
	PAT	10	10	1.000	10	10	1.000
	PCO	0	0	+++	0	0	+++
	PPC	0	0	+++	0	0	+++
	PAR	0	0	+++	0	0	+++
	PREP	0	0	+++	0	0	+++
	PRES	0	0	+++	0	0	+++
	PES	0	0	+++	0	0	+++
	PDS	0	0	+++	0	0	+++
	PRS	0	0	+++	0	0	+++
	PS	1	1	1.000	1	1	1.000
	PWA	0	0	+++	0	0	+++
	Opp T	1	1	1.000	1	1	1.000
	RT	2	2	1.000	2	2	1.000
	N/C	31	31	1.000	31	31	1.000
	IND	0	0	+++	0	0	+++

*+ Occurrences number = 146; +++ Categories without values were not coded by the observers. Evaluative + (EV+); Evaluative – (EV−); Descriptive (DES); Prescriptive (PRE); Interrogative (INT); Affectivity + (AF+); Affectivity – (AF−); Hearing (H); Visual (VIS); Hearing-visual (H-VIS); Athlete (ATL); Substitute Athlete (SA); Group (GRO): Defenses Group (DG); Midfielders Group (MG); Forward Group (FG); Substitutes Group (SG); Team (T); Technique (TEC); Offensive Techniques (OFTE); Defensive Techniques (DEFTE); Tactic (TAT); Game System (TAGS); Game Methods (TAGM); Tactical Schemes (TATS); Game Principles (TAGP); Functions/Missions (TAFUNC); Combinations (TACOMB); General Effectiveness (TAGE); Psychological (PSY); Game Rhythm (PGR); Confidence (PC); Pressure effectiveness (PPE); Attention (PAT); Attention (PCO); Combative pressure (PPC); Adversity resistance (PAR); Responsibility (PRE); Physical (FIS); Resistance (PRES); Execution speed (PES); Displacement speed (PDS); Reaction speed (PRS); Strength (PS); Warm-up (PWU); Opponent’s Team (Opp T); Referees Team (RT); No content (N/C); Indeterminate (IND).*

**TABLE 4:** Inter observer reliability of the observational system.

Criteria	Categories	Observer 1	Observer 2	Valor de Kappa
Objective (+)	EV+	21	20	0.972
	EV−	2	2	1.000
	DES	6	5	0.906
	PRE	111	112	0.981
	INT	2	2	1.000
	AF+	4	5	0.885
	AF−	0	0	+++
Form (+)	H	104	103	0.967
	VIS	0	0	+++
	H-AUVIS	42	43	0.967
Direction (+)	ATL	118	118	1.000
	SA	1	1	1.000
	DG	2	2	1.000
	MG	2	2	1.000
	FG	0	0	+++
	SG	4	4	1.000
	T	19	19	1.000
Content (+)	OFTE	6	6	1.000
	DEFTE	0	0	+++
	TAGS	0	0	+++
	TAGM	25	25	0.952
	TATS	32	33	0.942
	TAGP	3	3	1.000
	TAFUN	3	3	1.000
	TACOM	0	0	+++
	TAGE	3	3	1.000
	PGR	2	2	1.000
	PC	0	0	+++
	PPE	27	26	0.931
	PAT	10	10	1.000
	PCO	0	0	+++
	PPC	0	0	+++
	PAR	0	0	+++
	PREP	0	0	+++
	PRES	0	0	+++
	PES	0	0	+++
	PDS	0	0	+++
	PRS	0	0	+++
	PS	1	1	1.000
	PWA	0	0	+++
	Opp T	1	1	1.000
	RT	2	2	1.000
	N/C	31	31	1.000
	IND	0	0	+++

*+ Occurrences number = 146; +++ Categories without values were not coded by the observers, with agreement and the value is constant. Evaluative + (EV+); Evaluative – (EV−); Descriptive (DES); Prescriptive (PRE); Interrogative (INT); Affectivity + (AF+); Affectivity – (AF−); Hearing (H); Visual (VIS); Hearing-visual (H-VIS); Athlete (ATL); Substitute Athlete (SA); Group (GRO): Defenses Group (DG); Midfielders Group (MG); Forward Group (FG); Substitutes Group (SG); Team (T); Technique (TEC); Offensive Techniques (OFTE); Defensive Techniques (DEFTE); Tactic (TAT); Game System (TAGS); Game Methods (TAGM); Tactical Schemes (TATS); Game Principles (TAGP); Functions/Missions (TAFUNC); Combinations (TACOMB); General Effectiveness (TAGE); Psychological (PSY); Game Rhythm (PGR); Confidence (PC); Pressure effectiveness (PPE); Attention (PAT); Attention (PCO); Combative pressure (PPC); Adversity resistance (PAR); Responsibility (PRE); Physical (FIS); Resistance (PRES); Execution speed (PES); Displacement speed (PDS); Reaction speed (PRS); Strength (PS); Warm-up (PWU); Opponent’s Team (Opp T); Referees Team (RT); No content (N/C); Indeterminate (IND).*

In Table 5 are shown the reliability coefficients of the Instruction Expectations in Competition Questionnaire. All correlation coefficients are significant at *p* ≤ 0.05, ranging between 0.825 (strong correlation) and 1 (perfect correlation). In Table 6 are shown the reliability coefficient of the Instruction Perception in Competition Questionnaire. In this analysis, all correlation coefficients were also significant at *p* ≤ 0.05 ranging between 0.884 (strong correlation) and 1 (perfect correlation).

**TABLE 5:** Reliability of the instruction expectations in competition questionnaire.

	Exp3 – Exp26	Exp6 – Exp22e	Exp13 – Exp22b	Exp14 – Exp21c	Exp24 – Exp20	Exp25 – Exp8	Exp28 – Exp5	Exp29 – Exp12
Correlation coefficient	0.825*	0.968*	0.896*	0.896*	0.976*	0.913*	0.968*	1.000*

*p ≤ 0.05.

**TABLE 6:** Reliability of the Instruction Perception in Competition Questionnaire.

	AutPerc3 – AutPerc26	AutPerc6 – AutPerc22e	AutPerc13 – AutPerc22b	AutPerc14 – AutPerc21c	AutPerc24 – AutPerc20	AutPerc25 – AutPerc8	AutPerc28 – AutPerc5	AutPerc29 – AutPerc12
Correlation coefficient	0.923*	1.000*	0.913*	0.943*	0.976*	1.000*	0.976*	0.884*

*p ≤ 0.05.

Table 7 are shown the results from the pilot study, in which expectations, instructional behavior, and perception of coaching behavior were recorded. Considering the proposed criteria, the coaches’ instructional behavior was preferentially prescriptive (*M* = 663.3), hearing (*M* = 491.5), directed to the individual (*M* = 701) and tactical content (*M* = 381.9). Considering the content, the most issued instruction was related to tactical schemes (*M* = 166.5). The coaches’ expectations about the instruction to be issued during the competitive moment were related to more positive affective information (*M* = 4.5), auditory-visual (*M* = 5), directed to the athlete (*M* = 4), group (*M* = 4), defenses (*M* = 4), and psychological combative pressure (*M* = 5). Regarding coaches’ self-perception about the instruction applied during the same competitive moment, behaviors were rated high on affective positive (*M* = 4.5), hearing (*M* = 4.5), directed to the athlete (*M* = 4), midfielders (*M* = 4), and team (*M* = 4), with psychological concentration content (*M* = 5).

**TABLE 7:** Pilot study: expectations – instructional behavior – self-perception.

Criterion	Categories	Expectations	Instructional behavior		Self-perception
				
			*M*	%	
Objective (+)	EV+	4	84.96	1.04	3.5
	EV−	2	19.89	2.35	1.5
	DES	2	35.79	4.23	2
	PRE	3	663.27	78.39	3.5
	INT	2	13.00	1.54	2
	AF+	4.5	28.75	3.40	4.5
	AF−	1	0.45	0.05	1
Form (+)	H	4	491.50	58.09	4.5
	VIS	3.5	3.00	0.35	3.5
	H-AUVIS	5	351.61	41.56	5
Direction (+)	ATL	4	701.00	82.85	4
	SA	2	28.80	3.40	2.5
	Group	4	37.02	4.37	3.5
	DG	4	13.11	1.55	2.5
	MG	3.5	15.68	1.85	4
	FG	2.5	2.11	0.25	3
	SG	2	6.13	0.72	2
	T	3.5	79.29	9.37	4
Content (+)	Technique	2	36.79	4.30	2.5
	OFTE	2	24.64	2.91	2.5
	DEFTE	3	11.75	1.39	3.5
	Tactical	3.5	381.88	45.13	3
	TAGS	3.5	2.77	2.45	3.5
	TAGM	3.5	117.71	18.91	2.5
	TATS	4	166.54	19.68	4.5
	TAGP	3.5	17.32	2.05	2.5
	TAFUN	3.5	26.89	3.18	4.5
	TACOM	2.5	14.11	1.67	2
	TAGE	3.5	18.54	2.19	4
	Psychological	5	231.25	27.33	4
	PGR	5	2.21	2.39	3.5
	PC	4.5	8.32	0.96	3.5
	PPE	5	129.61	15.32	4.5
	PAT	4.5	28.88	3.41	4.5
	PCO	4	0.55	0.07	5
	PPC	5	18.23	2.15	4.5
	PAR	4.5	23.34	2.76	3
	PREP	3.5	2.11	0.25	3
	Physical	3.5	15.54	1.84	3
	PRES	2	0.55	0.07	1.5
	PES	3	0.00	0.00	2.5
	PDS	4	0.00	0.00	3.5
	PRS	3.5	5.88	0.69	3
	PS	2.5	1.55	0.18	3
	PWA	2	7.55	0.89	2.5
	Opp T	4	4.45	0.53	3.5
	RT	3	2.55	2.43	4
	N/C	–	156.05	18.44	–

*Evaluative + (EV+); Evaluative – (EV−); Descriptive (DES); Prescriptive (PRE); Interrogative (INT); Affectivity + (AF+); Affectivity – (AF−); Hearing (H); Visual (VIS); Hearing-visual (H-VIS); Athlete (ATL); Substitute Athlete (SA); Group (GRO): Defenses Group (DG); Midfielders Group (MG); Forward Group (FG); Substitutes Group (SG); Team (T); Technique (TEC); Offensive Techniques (OFTE); Defensive Techniques (DEFTE); Tactic (TAT); Game System (TAGS); Game Methods (TAGM); Tactical Schemes (TATS); Game Principles (TAGP); Functions/Missions (TAFUNC); Combinations (TACOMB); General Effectiveness (TAGE); Psychological (PSY); Game Rhythm (PGR); Confidence (PC); Pressure effectiveness (PPE); Attention (PAT); Attention (PCO); Combative pressure (PPC); Adversity resistance (PAR); Responsibility (PRE); Physical (FIS); Resistance (PRES); Execution speed (PES); Displacement speed (PDS); Reaction speed (PRS); Strength (PS); Warm-up (PWU); Opponent’s Team (Opp T); Referees Team (RT); No content (N/C); Indeterminate (IND).*

## Discussion

The purpose of this study was to present the reliability of three validated measures, namely the System of Analysis of Instruction in Competition, the Questionnaire on Coach Instructional Behavior Expectations, and the Questionnaire on Coach Instructional Behavior Perception that could be used in a mix-method approach. The results related to intra-observer and inter-observer reliability showed that intra-observer reliability *k*-agreement values ranged between 0.912 and 1 for observer 1, and 0.82 and 1 for observer 2. For inter-observer reliability, *k*-agreement values ranged between 0.885 and 1 between observers. Thus, values for reliability are above acceptable. The correlation coefficient values recorded for the questionnaires on instruction expectations in competition were above 0.82 and for the questionnaire on instruction perception in competition above 0.88. These results were obtained after carefully examining the reliability of each instrument.

All instruments that were statistically examined can considered as reliable measures for coaching training programs, with a perspective of performance enhancement during competitive moments. Our findings for interrater reliability are comparable to those obtained for other observational system studies in the football context (e.g., Santos et al., 2012, 2014b). In addition, current results from the correlational analysis of the questionnaires are consistent with previous research considering these instruments (Santos and Rodrigues, 2006, 2019). Thus, the process of reliability analysis is crucial and determinant for instrument quality. The present results provide reliability of the instruments and thus can be used as measures of coaching behavior, that is concurrently associated with players and team performance (Choi et al., 2020). In this sense, the coach needs to think about the best communication strategies to maximize the players’ performance during competition (Kim and Park, 2020) and afterward have the ability to reflect on their behavior during the competitive moments. The coach behavior analysis model (Santos et al., 2019) can contribute to the development of coaching training programs, with the intuition of optimize the communication process. Given the importance of the coach intervention during competitive moments, the model proposed by Santos et al. (2019) provides clear guidance for coaches to prepare their intervention as a mean to increase athlete and overall team performance (Moen, 2014). Consequently, the measure of instructional behavior through the observational instrument, allows researchers better understanding of the coaches’ performance (Cope et al., 2017) and later perceiving their intervention to reflect on the instructional strategies used in the direction of the team during competition (Araya et al., 2015).

According to current results, there is little congruence between expectations, behaviors during competition, and self-perception meaning that there could be some variability among coaches. These results show that these instruments can aid coaches to reflect about their behaviors during competitive moments and to improve coach–athlete interactions, at a time of special difficulties, such as before competitive moments since there is variability according to these factors between coaches. These results are consistent with previous research (Keatlholetswe and Malete, 2019; Hague et al., 2021), as the cycle of expectations-behavior-perception/reflection can be determined for a more effective communication process, while bearing in mind the variability across coaches.

The added value of the mix-method approach allows researchers and practitioners to study coach instructional behavior in the context in which it is involved (competition) in depth, providing ecological validity (Portell et al., 2015; Anguera et al., 2018). Considering the validated observational instrument specifically for football, coaching behaviors are encoded with great reliability and thus present robust results regarding the application of coaching intervention during competitive moments (Anguera et al., 2018). The qualitative data collected as a result of the observation can be quantified or analyzed in a qualitative perspective using T-*patterns* and sequential analysis (Anguera et al., 2018). In addition, the validation and reliability process of the questionnaires allows researchers to mention that they effectively measure what they want to measure in a consistent way (Rhind et al., 2014).

### Limitations and Future Directions

Even though the current study shed some new insight related to coaching behavior and the mix-method approach in the football context, certain limitations should be acknowledged. First, the small number of coaches who participated in the pilot application. Although the sample was large enough to achieve adequate statistical power and collecting data from targeted samples such as sport coaches in the football context presents additional difficulties and barriers, especially considering these times of COVID pandemic, larger sample size may have yielded greater external reliability. On the other hand, the competitive context of football should not be extrapolated to other contexts where competition is of greater importance or where training and learning are much more relevant. Therefore, we suggest that mix-method studies should be developed in the diverse contexts of sport and especially in football with young athletes. Also, future studies can be carried out, in a prolonged period of the sports season, to apply this mix-method approach, thus evaluating progress in the communication process during competition. We suggest the continuous development of the study regarding the metric qualities of the instruments. Hence, future studies are welcomed with more robust tests that can be carried out favoring greater validity in their application.

## Conclusion

The three instruments displayed adequate reliability to be used concurrently during a competitive moment, as a mean to measure expectations, behaviors and perpections of coaching behavior. The mixed-method approach on coaches’ behaviors could be an interesting approach on football research, specifically those related to coaching behavior, and the coach training, education, and development could benefit from this type of assessment. These instruments proposed in the study constitute the basis for coaches training program considering their professional intervention in football. While we do not suggest that the mixed method approach outlined in this study should be the only one for studying coaches’ expectations and perceptions, it offers a possible and comprehensive approach to study coaching behavior processes and is particularly useful for studying specific coaching conducts in youth football. Each coaching process is unique, and it not easy to capture this contextual element and resultant situational effect within all competitive moments each coach goes through. This is particularly challenging in interpersonal coaching behavior.

### Practical Implications

The assessment of different coaching behaviors may prove fruitful for examining the role of coaches before, during, and after competition and their association with global functioning of athletes and team performance. More specifically, the inclusion of a mix-method analysis can be helpful to examine the extent to which coaches show higher levels of expectations or reflections as a mean to attempt to regulate their behaviors toward coaching during a competitive setting. These reliable instruments could also be used to examine if coaches with high/low levels of expectations and reflections show more resilience in face of adversity (e.g., defeat or a set-back in a performance), or show more positive outcomes (e.g., more concentration, better performance, and sustained engagement) and less negative psychological outcomes.

## Data Availability Statement

The raw data supporting the conclusions of this article will be made available by the corresponding author, upon request.

## Ethics Statement

The studies involving human participants were reviewed and approved by the Madeira University. The patients/participants provided their written informed consent to participate in this study.

## Author Contributions

FS and JR conceived this manuscript, led the writing team, conducted the study search, summarized the quantitative review, and drafted the “Results” section. ME and RR made substantial contributions to the “Discussion” section. FR revised the entire manuscript and made important contributions in various sections. All authors read and approved the final version of the manuscript.

## Conflict of Interest

The authors declare that the research was conducted in the absence of any commercial or financial relationships that could be construed as a potential conflict of interest.
